# Long-term care use among people living with dementia: a retrospective register-based study from Sweden

**DOI:** 10.1186/s12877-022-03713-0

**Published:** 2022-12-27

**Authors:** Atiqur sm-Rahman, Bettina Meinow, Lars-Christer Hydén, Susanne Kelfve

**Affiliations:** 1 Department of Culture and Society (IKOS), Division Ageing and Social Change (ASC), Linköping University, Norrköping, Sweden; 2 Aging Research Center, Department of Neurobiology, Care Sciences and Society, Karolinska Institutet and Stockholm University, Solna, Sweden; 3 Stockholm Gerontology Research Center, Stockholm, Sweden; 4 Center for Dementia Research (CEDER), Linköping University, Norrköping, Sweden; 5 Department of Culture and Society (IKOS), Division Social Work (SOCARB), Linköping University, Norrköping, Sweden

**Keywords:** Dementia, Long-term care, Use of eldercare, End-of-life, Register data, Sweden

## Abstract

**Background:**

Although many people with dementia need progressive support during their last years of life little is known to what extent they use formal long-term care (LTC). This study investigates the use of LTC, including residential care and homecare, in the month preceding death, as well as the number of months spent in residential care, among Swedish older decedents with a dementia diagnosis, compared with those without a dementia diagnosis.

**Methodology:**

This retrospective cohort study identified all people who died in November 2019 in Sweden aged 70 years and older (*n* = 6294). Dementia diagnoses were collected from the National Patient Register (before death) and the National Cause of Death Register (death certificate). The use of LTC was based on the Social Services Register and sociodemographic factors were provided by Statistics Sweden. We performed regression models (multinomial and linear logistic regression models) to examine the association between the utilization of LTC and the independent variables.

**Results:**

Not only dementia diagnosis but also time spent with the diagnosis was crucial for the use of LTC in the month preceding death, in particular residential care. Three out of four of the decedents with dementia and one fourth of those without dementia lived in a residential care facility in the month preceding death. People who were diagnosed more recently were more likely to use homecare (e.g., diagnosis for 1 year or less: home care 29%, residential care 56%), while the predicted proportion of using residential care increased substantially for those who had lived longer with a diagnosis (e.g., diagnosis for 7 + years: home care 11%, residential care 85%). On average, people with a dementia diagnosis stayed six months longer in residential care, compared with people without a diagnosis.

**Conclusions:**

People living with dementia use more LTC and spend longer time in residential care than those without dementia. The use of LTC is primarily influenced by the time with a dementia diagnosis. Our study suggests conducting more research to investigate differences between people living with different dementia diagnoses with co-morbidities.

## Background

Dementia is a major public health concern that affects about 55 million people worldwide and is expected to triple by 2050 [1]. Although many people living with a dementia diagnosis (PlwD) can live on their own in the early and mid-phases of the disorder, the progressive nature of the disease may bring changes in an individual’s life at different stages with an increased need for support [2–4]. That means PlwD often have functional, cognitive, and communicative disabilities and may have challenges performing everyday tasks during the course of the disease [5–7]. Thus, especially during the last years of life, people with dementia have an increasing demand for long-term care (LTC; including homecare and residential care) [8, 9].

Due to different systems of LTC, given a certain level of care needs, the use of services varies across countries [10, 11]. In the Nordic countries LTC is largely publicly financed with the goal of an equitable allocation of care on the basis of universalism [12], e.g., access to LTC is needs-tested rather than means-tested. Although aging-in-place, e.g., to assist older people in their own homes, is promoted in all Nordic countries [13, 14], the use of LTC depends on the country-specific welfare system. Although several European countries impose legal obligation to family members to provide care support to older adults [15], in the Nordic countries, there is no formal obligation nor strong norms for the family to care for older parents [12, 16].

Sweden has an established care system, an integrated part of the universal welfare system, that provides both health and social care services for older adults aged 65 + years. The 290 Swedish municipalities are legally obliged to provide LTC services, which are largely financed by local taxes and services are used by all socioeconomic groups. Individual income-related user fees are low, with a cap of currently ~ 225$ per month, covering 4–5% of the actual costs [17]. A needs assessor, deployed by the municipality makes a need assessment and decides whether a person will receive services, and if so what kind and how much. However, although access to LTC is needs-tested, due to the local autonomy of the municipalities, there are no uniform rules that specify the amount of help to which a person is entitled to, given a certain degree of dependency. Home care and residential care are the main forms of publicly provided LTC services [18, 19]. Home care includes both assistance with household tasks (e.g., shopping, cleaning house, meals on wheels or cooking, doing laundry) and help with personal care (e.g., bathing/ showering, toileting, dressing, eating) [20, 21]. Residential care comprises LTC facilities where the older person has a small apartment with access to (unlimited) care and services available around the clock and, in some municipalities, sheltered accommodation providing specified and individually granted care and services.

In Sweden, nearly 60% of people living with a dementia diagnosis are living at home while 42% live in residential care facilities [22, 23], and an estimated 24% percent of all persons living with a dementia diagnosis in Sweden do not use any LTC [20, 24, 25]. A closer analysis of those without any LTC indicates that sociodemographic factors such as age, gender, education, whether a person lives together with someone or not, and time with a dementia diagnosis are important factors associated with the use or non-use of LTC [24, 26].

Previous studies on the use of LTC among PlwD were restricted focused primarily on local data [17, 27] and provided to cross-sectional snapshots [28, 29]. Moreover, it is unclear for how long people with dementia live in residential care after being diagnosed. In this study, we start in the month preceding death and follow the use of LTC services retrospectively. Thus, we track the use of LTC during the period of life when care needs accelerate. To predict the future needs for health and social care for people living with a dementia diagnosis and to further develop policies around LTC for people with dementia, it is important to know to what extent they use LTC, especially during the last years of life, and if and how social factors influence the use.

## Research aim and questions

The overarching aim of this study is to investigate the use of LTC among Swedish older adults aged 70 + with a dementia diagnosis. To better understand the use of LTC among people with a dementia diagnosis, we compare this group with those without a dementia diagnosis. More specifically three research questions are posed:

- [1] Which type of LTC (no care, home care, residential care) did people aged 70+ with a dementia diagnosis use in the month preceding death compared to those without a dementia diagnosis?
- [2] What sociodemographic factors (age, gender, education, cohabitation status) were associated with the use of LTC among people with dementia, compared with people without dementia? And how was the time with dementia diagnosis associated with the use of LTC?
- [3] For how many months did people with dementia live in residential care? How were sociodemographic factors (age, gender, education, cohabitation status) and the time with dementia diagnosis related to the length of stay?

## Methods

### Study design

This was a secondary data analysis of a retrospective cohort study.

### Data sources

Data were derived from four different Swedish registers linking the Longitudinal Integration Database for Health Insurance and Labour Market Studies (LISA), with the National Cause of Death Register (CDR), the National Patient Registers (NPR), and the Social Service Register (SSR). Information on sociodemographic factors was extracted from LISA administered by Statistics Sweden. Dementia cases were determined in the CDR and in the NPR, based on International Classification of Disease (ICD-10) codes [30], considering all primary and secondary diagnoses or causes of death. The CDR provides dementia diagnosis in the death certificate. Information on an individual’s death is recorded in the CDR based on a two-stage death certification. First, as soon as a physician confirms a death, it is immediately reported to the Swedish Tax Agency called ‘notification of death’ (*dödsbevis* in Swedish) that must be completed before burial. Second, another report, called the medical death certificate (*intyg om dödsorsaken* in Swedish), has to be sent to the National Board of Health and Welfare within 3 weeks of the death [31]. Data on dementia diagnosis before death came from the NPR (in-patient care as well as specialist care). Both CDR and NPR are administered by the National Board of Health and Welfare. The record of the use of LTC was retrieved from the SSR database, that gathers monthly data on granted and provided LTC, also administered by the National Board of Health and Welfare. A unique personal identification number for each inhabitant in Sweden facilitated the linkage of the different registers on the individual level.

### Study population

The study population included all older adults living in Sweden, who died in November 2019 aged 70 years and older. As 65 years is the minimum age for being eligible for LTC for older people and individuals were followed retrospectively for the last five years of life, only those aged 70 years and over at the time of death in 2019 were included (n = 6294). To analyse factors associated with the use of LTC we compared three groups: people who were never diagnosed with dementia, people diagnosed with dementia before death, and people diagnosed with dementia in the death certificate. The dementia diagnosis was following ICD10-codes (F00-F03 or G30-G32) using NPR (before death) and CDR (in death certificate). More than half of all PlwD’s diagnosis was recorded in both NPR and CDR registers, almost one fourth were diagnosed in death certificate and less than a quarter was diagnosed before death.

## Measures

### Outcome measures

Use of long-term care, i.e., home care or residential care, was measured in the month preceding death (October 2019). Time spent in residential care was measured monthly for five years (60 months) before death, ranging from 1–60 months. This means that people with ‘60 months’ in residential care may have lived there 60 months or more.

### Covariates

We considered the following socio-demographic covariates: *sex* and *age at death* (grouped in six age classes- 70 to 74 years, 75 to 79 years, 80 to 85 years, 86 to 89 years, 90 to 95 years, and 95 + years); *Education* was measured as the highest level of education and categorized into three levels as compulsory, secondary, and tertiary; people with missing information on education (*n* = 92) were considered in a separate category. *Cohabitation status* was classified as cohabiting (married or share household) or living alone.

*Time with dementia diagnosis,* calculated in years from the first time the person was diagnosed with dementia in any of the registers, was categorized as diagnosis at postmortem (in death certificate), 1 year or less, 2–3 years, 4–6 years, and 7+ years. We considered the latest data available for cohabitation status (November 2018) and for type of LTC (October 2019).

### Statistical analysis

Descriptive statistics were performed separately for people with and without dementia to describe socio-demographic characteristics of the study population and their use of LTC. Age at death was presented with means and standard deviations and time with dementia diagnosis was presented with percentage. To model the association between the utilization of LTC and the independent variables (time of dementia diagnosis, age, gender, and cohabitation status), we used multinomial and linear logistic regression analyses [32]. We estimated predicted proportions with LTC and the predicted number of months in residential care facilities (calculated from the margins command) for people with and without a dementia diagnosis. We used both bivariate and adjusted models for all predictors. All statistical analyses were performed in STATA 14 (StataCorp, College Station, TX).

## Results

### Participants

The study population (*n* = 6294) comprised two groups: (1) people living with dementia (*n* = 1821) and (2) people without dementia (*n* = 4473). We consider the total population at each stage of the analysis.

### Descriptive data

Of all individuals aged 70 + who died during November 2019, 29% had a dementia diagnosis (Table 1). Of these, about a quarter were diagnosed with dementia after death (at death certificate). Compared to persons without a dementia diagnosis, those with a diagnosis were older (mean age 83.6, SD = 8.0 vs. mean age 86.9, SD = 6.5), and more often women (60%). About three quarters of people with a dementia diagnosis were living alone in the month preceding death compared to two thirds of those without a dementia diagnosis. People with dementia more commonly used LTC in the month preceding death compared with those without a diagnosis (92% vs. 59%). In the month preceding death, three of four people with a dementia diagnosis lived in residential care, while one of four lived at home– either with home care (18%) or without using any LTC (7%). The mean time spent in residential care was two and a half years for people with a dementia diagnosis and two years for those without a diagnosis. Since the educational level was similar among people with and without dementia, with almost 50% with primary education, we excluded education from further analyses.

**Table 1:** Description of the study population aged 70 + with and without a dementia (PlwithoutD) diagnosis

	**PlwD** ***N***(%)	**PlwithoutD** ***N***(%)	**Total** ***N***(%)
*Sex*	*n* = 1821 (29%)	*n* = 4473 (71%)	*n* = 6294
Men	727(39.9)	2229(49.8)	2956(47.0)
Women	1094(60.1)	2244(50.2)	3338(53.0)
*Age at death (year)*			
Mean (SD)	86.9 (6.5)	83.6 (8.0)	84.6 (7.7)
70–74	83(4.6)	706(15.8)	789(12.5)
75–79	179(9.8)	811(18.1)	990(15.7)
80–84	343(18.8)	868(19.4)	1211(19.2)
85–89	515(28.3)	930(20.8)	1445(23.0)
90–94	505(27.7)	729(16.3)	1234(19.6)
95 +	196(10.8)	429(9.6)	625(9.9)
*Education*			
Compulsory	893(49.0)	2111(47.2)	3004(47.7)
Secondary	608(33.4)	1603(35.8)	2211(35.1)
Tertiary	288(15.8)	699(15.6)	987(15.7)
Missing information	32(1.8)	60(1.3)	92(1.5)
*Cohabitation status in the month preceding death*			
Living alone	1355(74.4)	3010(67.3)	4365(69.4)
Cohabiting	466(25.6)	1463(32.7)	1929(30.7)
*Time with dementia diagnosis*			
No dementia	0	4473(100)	4473(71.1)
Diagnosed in death certificate	426(23.4)	0	426(6.8)
1 year or less	416(22.8)	0	416(6.6)
2–3 years	369(20.3)	0	369(5.9)
4–6 years	361(19.8)	0	361(5.7)
7 + years	249(13.7)	0	249(4.0)
*LTC in the month preceding death*			
No care	141(7.7)	1817(40.6)	1958(31.1)
Home care	319(17.5)	1635(36.5)	1954(31.0)
Residential care	1361(74.7)	1021(22.8)	2382(37.8)
*Months in residential care*			
Md (IQR)	28 (36)	18 (36)	23 (37)
10p	4	2	3
25p	12	6	9
75p	48	42	46
90p	60	60	60

### Outcome and main results

Table 2 presents an analysis of the predicted proportions of three groups of LTC users: no care, homecare, residential care, and to what extent sociodemographic factors (age, sex, cohabitation) and the time spent with a dementia diagnosis were associated with LTC use in the month preceding death. Results show that it was primarily time with a dementia diagnosis that affected the type of LTC a person used. For example, in the adjusted model, the predicted proportion of using LTC, in most cases residential care, was higher among people who had a dementia diagnosis for a longer time (e.g., 7 + years: homecare 11%, residential care 85%) compared with those who were diagnosed more recently (e.g., 1 year or less: homecare 29%, residential care 56%).

**Table 2:** Predicted proportion of LTC use for people aged 70 + with and without a dementia diagnosis in the month preceding death by time of dementia diagnosis, age group, sex, and cohabitation status

	No care			Homecare			Residential care		
	RRR	*p-values*	Predicted prop	RRR	*p-values*	Predicted prop	RRR	*p-values*	Predicted prop
**Adjusted**									
No dementia	ref		37.89	ref		37.21	ref		24.91
In death certificate			13.91	1.40	0.147	14.61	12.58	0.000	71.48
1 year or less			14.94	2.43	0.000	28.71	8.40	0.000	56.36
2–3 years			8.60	2.91	0.000	18.14	22.11	0.000	73.26
4–6 years			7.70	2.41	0.001	13.11	27.85	0.000	79.19
7 + years			3.64	4.39	0.001	10.78	68.76	0.000	85.58
*Age*									
70–74	ref		52.10	ref		25.85	ref		22.05
75–79			43.03	1.34	0.009	27.35	2.02	0.000	29.63
80–84			33.12	2.14	0.000	32.45	3.51	0.000	34.43
85–89			25.09	3.22	0.000	36.05	5.84	0.000	38.86
90–94			16.14	5.21	0.000	36.02	13.13	0.000	47.83
95 +			11.64	5.76	0.000	27.71	26.97	0.000	60.65
*Sex*									
Men	ref		33.64	ref		30.09	ref		36.26
Women			27.84	1.34	0.000	31.65	1.56	0.000	40.51
*Cohabitation status*									
Cohabiting	ref		39.09	ref		31.03	ref		29.88
Living alone			26.62	1.67	0.000	31.27	2.99	0.000	42.12

Notably, in the adjusted model, the predicted proportion of those who did not use any LTC care was considerably higher among individuals who were diagnosed with dementia after death (in the death certificate). Regarding different age groups, younger decedents with a dementia diagnosis (e.g., 75–79 years) were less likely to have used any kind of LTC care (43%) compared to other older age groups. In the month preceding death, residential care was somewhat more common among women (41%) than men (36%) and among individuals living alone (42%) compared to cohabiting people (30%). An equal share of people without dementia diagnosis had died either with home care (37%) or without using any kind of LTC (38%).

Based on linear regression models, Table 3 shows the predicted number of months with residential care among decedents aged 70 + with and without dementia. The predicted number of months living in a residential care facility was higher for people who had spent more years with a dementia diagnosis. For example, people with 7 + years with a dementia diagnosis spent longer time (46 months) in residential care compared with those who had the diagnosis for 1 year or less (18.5 months). This was also true for the oldest age group (e.g., 95 + year: 33 months) who spent longer time in residential care compared with the younger age group (e.g., 75–79 years: 24 months). The predicted number of months spent in residential care was higher for women (30 months) compared with men (24 months) and for those living alone (30 months) compared with cohabiting people (18 months).

**Table 3:** Predicted number of months in residential care for people aged 70 + with and without dementia in the month preceding death by time with dementia diagnosis, age group, sex, and cohabitation status

	**Bivariate**			**Adjusted**		
	Coef	*p-values*	Predicted number of months	Coef	*p-values*	Predicted number of months
*Time with dementia diagnosis*						
No dementia	ref		24.46	ref		23.60
Diagnosed in death certificate	5.41	0.000	29.87	5.94	0.000	19.71
1 year or less	-5.88	0.000	18.58	-3.89	0.004	25.61
2–3 years	0.35	0.788	24.81	2.01	0.110	34.96
4–6 years	9.20	0.000	33.66	11.36	0.000	46.59
7 + years	21.55	0.000	46.01	22.99	0.000	29.54
*Age*						
70–74	ref		27.27	ref		27.23
75–79	-2.78	0.257	24.49	-0.83	0.710	26.40
80–84	-1.95	0.391	25.31	-1.28	0.536	25.95
85–89	-0.50	0.819	26.77	-0.83	0.680	26.40
90–94	0.41	0.852	27.67	0.21	0.917	27.44
95 +	6.11	0.007	33.38	5.45	0.009	32.69
*Sex*						
Men	ref		24.26	ref		26.55
Women	5.49	0.000	29.75	1.86	0.024	28.41
*Cohabitation status*						
Cohabiting	ref		17.72	ref		18.36
Living alone	12.27	0.000	30.00	11.49	0.000	29.85

Focusing on differences across sociodemographic factors, we adjusted the analyses for age, sex, and cohabitation status. In the adjusted model, the predicted number of months spent in residential care increased for people with a shorter time with dementia diagnosis (e.g., 1 year or less: from 18.5 to 25.6 months) and was consistent up to six years. The number of months spent in residential care was lower for those with the longest time with diagnosis (7 + years: from 46 to 29.5 months) and for those who got their diagnosis in the death certificate (from 29.8 to 19.7 months). We did not find any significant age, sex, and cohabitation effect on using residential care in the adjusted model.

## Discussion

In this article, we analyzed national Swedish register data to investigate the use of LTC during the last five years of life among Swedish decedents aged 70 + with and without dementia diagnosis and how the use of LTC is associated with sociodemographic factors. The main result showed that PlwD used more LTC and spent longer time in residential care than those without dementia. In addition, the use of LTC was primarily influenced by the time with dementia diagnosis. In the following we discuss the main findings of our study.

1. Our results show that the most important factor for living in residential care at the end of life is the number of years with a dementia diagnosis. People in their early diagnosis period were more likely to use homecare (1 year or less: 29%), while the likelihood of using residential care was increased substantially for those with a longer time with dementia (7 + years: 86%). This may indicate that people with a long time with dementia diagnosis, on average, experienced more severe symptoms entailing a need for care and support around-the-clock. Contrasting to our findings, German [33] and Irish [4] studies suggested that the care situation at home, including caregivers’ socioeconomic status, is more important for the transition to residential care than for how long time an individual has had a dementia diagnosis or severity of impairments. Higher age, female sex, and multiple comorbidities were also identified as significant risk factors for a transition to residential care of PlwD [4, 34]. The fact that studies from different countries find different factors that are associated with moving to residential care, given a certain level of care needs, probably reflects the different systems for social care for older people, as well as differences in social policy in general, e.g., affecting the proportion of women in the labor market, which in turn affects their availability for informal care. The differences in study findings may also reflect differences in measures used and study populations including diversity of PlwD, dementia symptoms, types, and comorbidities.
2. On average, PlwD stayed six months longer in residential care, compared to the approximately two years among people without a diagnosis. This is similar to findings from Norway [3] and the Netherlands [34], where the median time in residential care for PlwD was 2.3 years and 2.5 years, respectively. The average length of stay in residential care facilities for older adults with dementia and other health conditions among six European countries was 2.4 years ranging from 1.8 years in Poland to 3.1 years in Belgium [35]. The availability of resources that allow older adults to stay longer at home and subsequently, delay the admission in LTC was found to be a commonly associated factor in most countries [35]. This may influence the length of stay in residential care. Besides that, differences in LTC utilization between different countries may be explained by country specific LTC management, access to residential care, older age at admission, impairment in performing activities of daily living (ADL), and comorbidities.
3. In our study, the only sociodemographic factor that showed an association with the length of stay in residential care was cohabitation status: people living alone spent longer time in residential care compared to those cohabiting. This indicates that the potential access to informal care from a spouse may have postponed a move to residential care. Similarly, in Belgium and in Finland, living with partner or being married was associated with a shorter stay in residential care among PlwD [35]. It is highly likely for married or cohabiting PlwD to stay longer time at home before entering residential care. Although cohabitation plays a crucial role for the use of LTC, the capacity of caring for an older partner may be limited among very old couples, especially when care needs are extensive [19].

Our results further show that about one fourth of the people who died with dementia received their diagnosis in the death certificate. This may reflect underdiagnosis or recognition of dementia. It also indicates that dementia may not be the main reason for their move to residential care, but rather worsened conditions that engender extended and higher level of care needs. Previous studies acknowledged that the level of care needs (need of ADL support, impaired cognition, depression and agitation, frailty, falls) play a noteworthy role for moving to residential care [36, 37]. We found that about three quarters of all decedents with dementia, but only one fourth of those without a dementia diagnosis, used residential care facilities in the month preceding death. Several previous studies from Belgium, England, Scotland, the Netherlands, and the USA also demonstrated that the majority of PlwD lived in residential care at time of death [34, 38, 39].

## Strengths and limitations

We state that this study enhances understandings of LTC use among Swedish older adults aged 70 + who died with a dementia diagnosis and the factors associated with use of LTC. The main strength of this study is the combination of multiple Swedish registers that includes all Swedish decedents from one specific month without dropout [31, 40]. Previous registry based Swedish studies on the provision of LTC and length of stay mostly focused on different sociodemographic factors, living arrangement of older adults with dementia, and care situation at home. To the best of our knowledge, the present study is one of the first retrospective studies which examines the association between time with dementia diagnosis and the use of LTC along with sociodemographic predictors that captures 5 years of follow-up.

However, the study also has several limitations. First, we only have data about dementia diagnosis provided by hospital clinics but not about dementia diagnosis provided by General Practitioners in healthcare centers. Several previous studies have reported that dementia diagnosis in Swedish registers varied with regards to average age of onset, likelihood of capturing various types of dementia or different severity of the disease [40–42]. For example, while 7.8% of the people in the National Inpatient register were diagnosed with dementia at an average age of 83.5 (SD 5.3), the National Outpatient register (5.1%, average age 89.0 years, SD 5.6) and the CDR (8.1%, average age 85.3 years, SD 5.1) showed slightly different figures. Therefore, it is plausible that some individuals with a dementia diagnosis may be missing in our data. However, a recent validation study showed that the detection rate for dementia diagnosis in NPR and CDR have nearly perfect specificity (99.8% for NIPR and 99.0% for CDR) [40]. Second, we did not have access to measures of frailty, functional status, or socioeconomic status as these are not recorded in national registers including all inhabitants. Finally, the time with dementia diagnosis may be underestimated since we do not have data from primary care.

## Conclusions

In conclusion, results showed that not only the dementia diagnosis but also the years spent with the diagnosis influence the use of LTC, specifically residential care. Moreover, cohabitation also affected the length of stay in residential care. We recommend conducting more and further research into this area. It might for instance be of interest to investigate differences between people living with different dementia diagnoses and associated co-morbidities. The findings further suggest that LTC providers in Sweden may need to extend the existing around-the-clock care assistance for home dwelling people with dementia offering more day care services, continuous supervision, longer home care visits, and possible technological assistance.

## Data Availability

The datasets used in the current study are not publicly available, in accordance with specific privacy restrictions. The datasets are stored at the Division Ageing and Social Change at Linköping University. Requests for access to the data that support the findings of this study can be put to the Head of Division, Division Ageing and Social Change, LiU (andreas.motelklingebiel@liu.se) and will be handled according to the relevant legislation.

## Abbreviations

PlwD: People living with Dementia
LISA: Longitudinal integrated database for health insurance and labour market studies
NPR: National Patient Register
CDR: National Cause of Death Register
SSR: Social Service Register
RRR: Relative Risk Ratios
SD: Standard Deviation
ADL: Activities of Daily Living
